# Glass Pessary Broken in the Vagina

**Published:** 1846-03

**Authors:** 


### *Glass Pessary Broken in the Vagina.*

*January* 14, 1846. Was called early this morning to visit Miss R. E. The patient is a maiden lady of about 40, and has been troubled for several years with prolapsus uteri, and for a long time has been obliged to wear a pessary. Those used have generally been of the gum elastic kind, which after being worn for two or three months, it was necessary to remove to cleanse, as the secretions which gathered about them became too irritating to bear. On this account, after attending to the removal and introduction of the instrument several times, I recommended the use of the *glass* pessary, as less irritating, less liable to collect the secretions, and more durable. Accordingly I procured and introduced one of size No. 2, Aug. 21, 1845. It answered the end designed, and gave rise to no inconvenience or trouble till this date. On arriving at her residence, she said she had not sent for me because she was sick, but because she was "scared." Inquiring the cause of her alarm, she told me that the pessary had broken—that while standing at the window, doing nothing, she heard a noise, and that any effort since had caused pain as of something pricking her. She could not account for it, unless, as she humorously remarked, it was *frosty!*—it being a cold morning. On examination I found it broken, indeed, into a great number of pieces. Parts of the periphery were in situ, and all the parts were at the upper part of the vagina. I found I had an unenviable task before me—the extraction of these sharp angular and pointed pieces of glass from the vagina, lined with a delicate mucous membrane, lying in rugæ. I had some doubt of the feasibility of the operation, and some apprehension for the result. But I commenced operations, and after two hours and a half of diligent and most careful manipulations, I succeeded in extracting every vestige of the glass. At least, several examinations afterwards, in several positions of the patient, did not discover the least particle remaining. Notwithstanding the care used, however, the vagina was unavoidably somewhat lacerated, so that a little hemorrhage was produced. Perhaps a tablespoonful of blood accompanied and followed the operation. My own fingers were also cut a little. The central piece, which was entire and averaging 1½ inch in diameter, having two rims, of which the edges were very jagged and pointed, was the most difficult to extract. Fearing that severe inflammation might ensue, I prescribed the antiphlogistic treatment and regimen, and an opiate and astringent injection to be frequently thrown into the vagina.

16th.—A slight fever ensued, considerable pain of bowels, especially in moving. Also some dysuria and tenesmus were present. Patient complained of a sensation of pricking, which was supposed to depend upon a small piece of glass remaining. A most careful examination, however, did not discover any such thing. The antiphlogistic treatment and injections were continued. The prospect at present (Jan. 20) is that the affair will not result in anything serious.

The patient had a severe fall on the fundament about ten days previous, but the absence of any pain or uneasiness from the pessary would lead to the conclusion that the instrument could not have been broken at that time. The number of pieces of glass extracted was fifty, of all shapes and angles.

The inference from the above is very plain—that there is danger attending the use of glass pessaries. In future, in my own practice, I think I shall not employ them, so long as others can be obtained.—W.

*Boston Med. and Surg. Journ.*

